# Competencies for bioinformatics core facility scientists: extension of the ISCB competency framework for bioinformatics

**DOI:** 10.1093/bioadv/vbaf206

**Published:** 2025-09-16

**Authors:** Marta Lloret-Llinares, Patricia Carvajal-López, Tim Downing, Madelaine Gogol, Shannan Ho Sui, Lara Nonell, Lisanna Paladin, Adam James Reid, Gina-Maria Pomann, Cath Brooksbank

**Affiliations:** EMBL’s European Bioinformatics Institute, Wellcome Genome Campus, Hinxton, Cambridge CB10 1SD, United Kingdom; EMBL’s European Bioinformatics Institute, Wellcome Genome Campus, Hinxton, Cambridge CB10 1SD, United Kingdom; Pirbright Institute, Woking, GU24 0NF, United Kingdom; Stowers Institute, Kansas City, MO 64110, United States; Harvard T.H. Chan School of Public Health, Boston, MA 02115, United States; Vall d’Hebron Institute of Oncology, Barcelona, 08035, Spain; European Molecular Biology Laboratory, Heidelberg, 69117, Germany; Gurdon Institute, University of Cambridge, Cambridge, CB2 1QN, United Kingdom; Duke University Department of Biostatistics and Bioinformatics, Clinical Translational Science Institute Biostatistics Epidemiology and Research Design Methods Core, Durham, NC 27705, United States; EMBL’s European Bioinformatics Institute, Wellcome Genome Campus, Hinxton, Cambridge CB10 1SD, United Kingdom

## Abstract

**Motivation:**

The competency framework of the International Society of Computational Biology (ISCB) provides a benchmark for capturing the knowledge, skills, and attitudes required by bioinformatics professionals. Whilst it provides a minimum standard for various bioinformatics roles, it does not capture how competency requirements change as bioinformatics professionals progress from junior to senior, and from primarily technical to managerial positions. Bioinformatics core facility professionals are crucial for data-driven bioscience, yet lack a defined career structure, leaving a career-development vacuum. The ISCB education and BioInfoCore communities worked together to define a new subset of competency requirements to support core facility teams to recruit, develop, and retain their staff.

**Results:**

Drawing on the experience of the ISCB’s BioInfoCore community, and building on the work of others, we extend the competencies required by staff in bioinformatics core facilities operations and management, defining six competency requirements (in addition to 13 defined in the ISCB Competency Framework) that are especially relevant to core facility professionals: N: identify and support users’ needs, O: manage projects, P: manage team members, Q: engage with users and other collaborators, R: provide training in bioinformatics, and S: lead the bioinformatics core facility. We map these to a framework for career progression.

**Availability and implementation:**

The framework is reproduced in full in this paper and is available as a CSV file on Zenodo (DOI: 10.5281/zenodo.16630540). It will soon be made available on the Competency Hub at https://competency.ebi.ac.uk/. Data and figures are available on GitHub at https://github.com/downingtim/Competencies.

## 1 Introduction

*“To push the frontiers of knowledge and conquer some of the big questions in science, the RI staff of the future, such as facility managers and expert technicians, will need to be trained in the increasingly interdisciplinary crossroads between research and technology. These personnel provide expert advice to develop RI, support RI users and provide field-specific support. RI staff will also need to be supported with career pathways, recognition of their achievements, and positive employment and workplace conditions. RI workforce strategies will need to attract, retain, and train the uniquely skilled personnel who manage, maintain, and operate these facilities.”*

The need to develop and retain a workforce capable of delivering on national and international research infrastructure (RI) roadmaps is increasingly seen as a high priority for science funders and policymakers. This quotation from the 2024 Brisbane Statement, formulated at the 2024 International Conference on Research Infrastructures (ICRI 2024, https://icri2024.au/about-icri/brisbane-statement/) as a call to action for RI stakeholders, eloquently captures the rationale for the work described in this article.

Quantitative experts such as biostatisticians, bioinformaticians, epidemiologists, and other biodata specialists collaborate with research scientists, informing methods of data collection, design of experiments, appropriate quantitative analysis, visualization, interpretation, and dissemination of results. Such professionals may be embedded in individual research groups, in departmental or institutional core facilities (Turpen *et al.* 2016; Kumuthini *et al.* 2020), or—increasingly—in networked facilities, providing the RI essential for today’s large-scale, challenge-led, biological research.

The European Strategy Forum on Research Infrastructures (ESFRI), which was set up in 2002 to provide input to the European Commission on RI needs, has long considered the training and development of RI staff as a high priority. EC-funded projects such as CORBEL (EC grant agreement number 654248) and RItrain (EC grant agreement number 824087) laid the foundation for staff development in European RIs, both for technical staff (CORBEL, Brooksbank and Pasterk 2016, Lloret-Llinares *et al.* 2020; https://competency.ebi.ac.uk/framework/corbel/1.1) and for managers of RIs (RItrain, https://competency.ebi.ac.uk/framework/ritrain/1.0). In its 2020 White Paper (ESFRI 2021), ESFRI stated “The development of a formal curriculum for RI managers and its implementation in a dedicated master’s program has been an important milestone towards establishing a formal career path in RI management and fostering the general attractiveness of careers in the RI domain”; some ESFRI RIs, including ELIXIR, which is Europe’s distributed RI for biological data, have working groups dedicated to the professionalization of roles in RIs (https://elixir-europe.org/focus-groups/professionalising-careers#:∼:text=As%20a%20research%20infrastructure%2C%20ELIXIR,term%20of%20this%20focus%20group), Yet despite these encouraging signs, academic institutions find it increasingly challenging to recruit and retain RI staff. The need for better defined career pathways for these professionals, and for widespread recognition of them as peers and partners in research collaborations, is palpable: without it, the development of increasingly complex and expensive RI is futile. Policy briefings (e.g. Karoune and Sharan 2025) continue to urge policymakers to invest in the development of professional career structures in data science, demonstrating that this remains an unsolved problem.

Bioinformatics core facilities provide essential bioinformatics services such as advice on experimental design, high-throughput data analysis, training, data processing, exploratory data analysis, modelling molecular data, development of standardized analysis pipelines, data integration, and data visualization (Turpen *et al.* 2016; Kumuthini *et al.* 2020). Scientists in core facilities often require both biological subject-matter expertise (e.g. cancer or developmental biology) and advanced bioinformatics knowledge. Most hold advanced degrees (MSc, MRes, PhD) in the life sciences, computational sciences, statistics or bioinformatics, along with practical experience. Facilities are typically staffed by a manager who often also undertakes data analysis, and several junior and senior bioinformaticians with varied expertise taking on diverse projects (Dragon *et al.* 2020). Larger teams might have dedicated education and training staff, IT specialists, and statisticians, and there are varying degrees of integration with software engineering, scientific computing, DNA sequencing services, and other core facilities (e.g. imaging, mass spectrometry, and flow cytometry).

Increasingly, core facilities embedded within a single institution are networked nationally and internationally to form distributed international RIs supporting large-scale international projects. For example, in Europe, SciLifeLab (https://www.scilifelab.se/) leverages six institutions across Sweden to underpin cutting-edge molecular life-science research on a national scale; ELIXIR (https://elixir-europe.org/) provides bioinformatics services for Europe and beyond. In Australia, Australian BioCommons (https://www.biocommons.org.au/) supplies bioinformatics RI at a national level, whilst in Africa, the African Bioinformatics Institute (https://www.bioinformaticsinstitute.africa/) has recently been established to provide bioinformatics expertise, infrastructure, and resources across the continent. These large-scale initiatives have been seeded by smaller-scale core facilities. This important link between organizational and international RI is recognized by career development programs such as ARISE2 (https://www.embl.org/training/arise2/) led by the European Molecular Biology Laboratory, a 3-year fellowship program that trains its fellows in the development of technologies and methods for improvement of scientific services, while also preparing them for careers in RIs.

Core facilities provide distinct career tracks from classical academic roles, career-tracks that are frequently ill-defined (Lewis *et al.* 2016). Often they originate from specific institutional needs rather than structured professional pathways (Bartlett *et al.* 2017). As these collaboration- and service-oriented core facilities become more widespread and more interconnected, the need for professionals qualified to work in them increases, as does the need for a clear career structure to guide aspiring professionals that not only specifies the depth of expertise, knowledge and skills that bioinformaticians contribute, but also valorizes them as crucial contributors to bioscience teams and collaborations undertaking research on a previously unprecedented scale (Mulder *et al.* 2018). Despite their importance, many bioinformatics cores, particularly smaller ones, face operational challenges, including limited institutional support and reliance on cost-recovery models (Dragon *et al.* 2020). Professionals in bioinformatics cores have historically suffered from two stigmas that limit career progression: firstly, the stigma of working in an interdisciplinary field (Institute of Medicine (US) Committee on Building Bridges in the Brain, Behavioral, and Clinical Sciences 2000, Daniel *et al.* 2022), and secondly the stigma of working in a service setting rather than a research setting (Medical Research Council 2022).

Work has begun to define career pathways for core facilities roles. For biostatisticians, three main competency categories have been outlined: communication and leadership, domain expertise, and statistical expertise (Pomann *et al.* 2020, Slade *et al.* 2023, Peskoe *et al.* 2024). The Academic Data Science Alliance recently published a career guidebook for hiring managers, to support the retention of data scientists and research software engineers in academia (Van Tuyl 2023). Additionally, Global BioImaging published recommendations to support imaging scientists’ career progression and recognition within core facilities (Wright *et al.* 2024).

Yet despite these ongoing efforts, a significant gap remains in defining roles and career development pathways for bioinformatics core facility staff. This underscores the need for clear, structured competencies to ensure that bioinformatics staff can meet the diverse and evolving demands placed upon them; defining these roles adequately has become crucial for the future of bioinformatics in both academic and industrial settings.

The International Society for Computational Biology (ISCB) developed its bioinformatics competency framework in 2014 (Welch *et al.* 2014, Welch *et al.* 2016), partly with the goal of supporting employers who were struggling to recruit bioinformatics graduates with the necessary skill set. The framework has subsequently been revised twice (Mulder *et al.* 2018, Brooksbank *et al.* 2024). The third version includes a core facility scientist profile at an intermediate career stage, in addition to 14 other role profiles (https://competency.ebi.ac.uk/framework/iscb/3.0/career-profiles).

**Figure 1.**
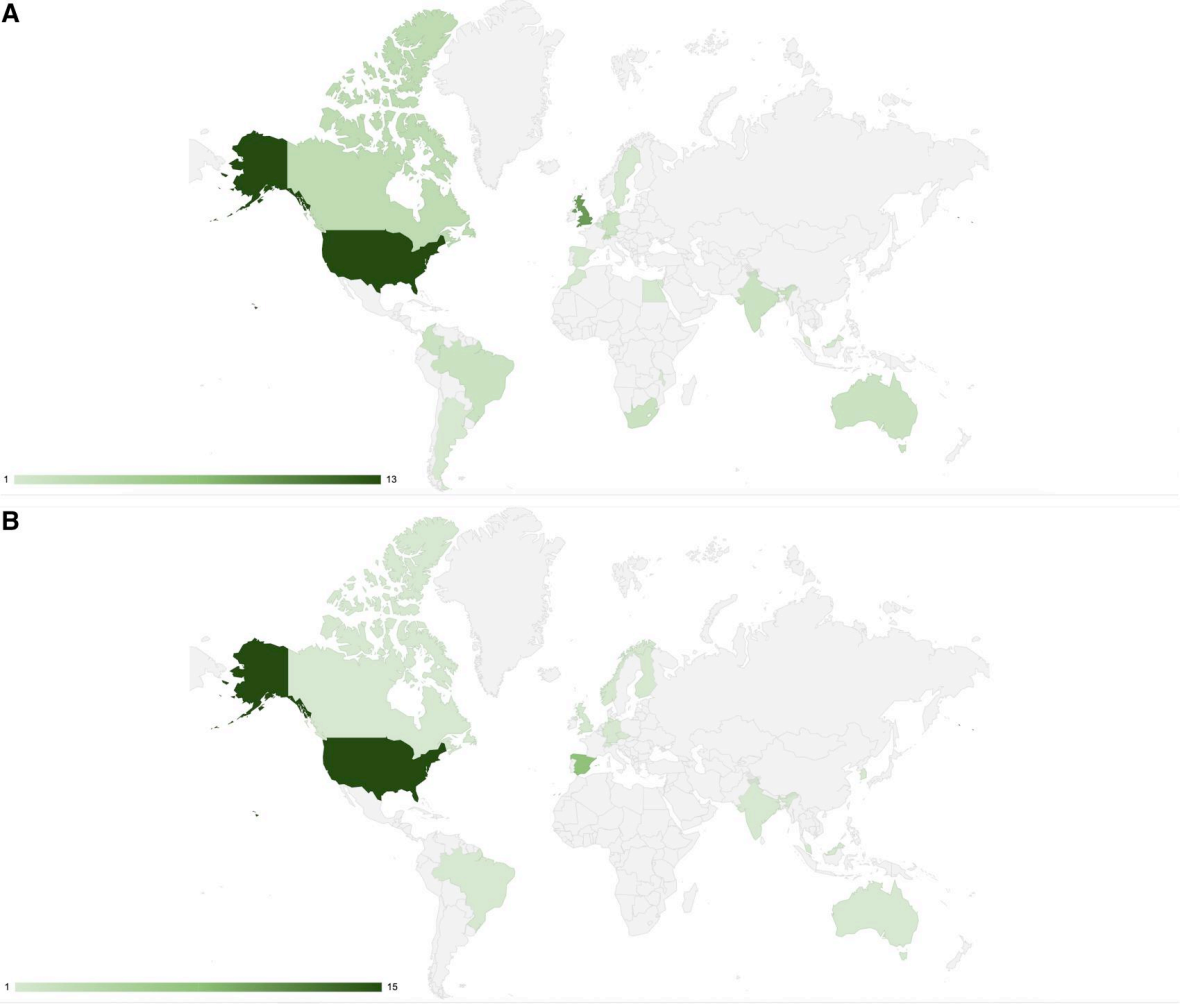
Geographical distribution of (A) participants in the working group that developed the new competencies for core facility scientists and (B) respondents to the survey. Survey responses provided the dataset for Figs 2–4.

We sought to extend the ISCB competency framework to represent the wide variety of roles in bioinformatics core facilities, through contributions from a working group of 48 members from 37 institutions across 17 countries (Fig. 1A). This revised framework defines three levels of core facility bioinformaticians (Scientist I, II, and III) and a managerial role (M). The framework is designed to be flexible and applicable in a variety of settings; it can be used as a starting point for defining roles tailored to specific needs.

This extension to the ISCB competency framework provides transparent career progression guidelines so that core facility bioinformaticians can readily identify and address their professional development needs. This illuminates clearer paths for individuals, potentially supporting improved staff retention and productivity within core facilities. Core facilities can offer interesting, stable, and highly rewarding careers and contribute valuable skills in academic environments where bioinformaticians not following traditional academic career paths might otherwise seek alternatives. We initially describe how the working group developed this framework, then we present the framework and how it can be used. We conclude by discussing existing gaps and future directions to further support this workforce and other bioinformatics professionals.

## 2 Methods

### 2.1 Defining competencies for professionals in bioinformatics core facilities

Version 3.0 of the ISCB competency framework defines a set of 13 competencies in the field of bioinformatics, which include knowledge, skills and attitudes to detail the level of competency [defined using Bloom’s Taxonomy (Anderson and Krathwohl 2001, Bloom *et al*. 1956)] required for students and professionals in the field (Mulder *et al.* 2018, Brooksbank *et al.* 2024). The framework includes a series of personas (also known as career profiles) that represent different roles in bioinformatics, with a short description of the background and the activities of the role together with the Bloom’s level at which each competency is required to work in that role. The focus of the ISCB competency framework is to enable educational institutions and training providers to design courses that develop relevant competencies in the field, so it describes each career profile as a minimum standard. It also supports employers to recognize the competencies missing in their teams and to take appropriate action, and it supports individual scientists to plan their professional development. Career progression is not reflected in version 3.0 of the framework, but there is a consensus among ISCB’s education community that stratifying roles into different career stages would support institutions to structure their workforce and guide the career development of individual professionals.

Input from a diverse range of professionals (see acknowledgements) working in bioinformatics core facilities was essential to extend the framework and define the competencies required by bioinformatics core facility staff, as well as to reflect changes in competency level with career progression. To reach out to this community, we ran two sessions during 2023, the first at the Global Bioinformatics Education Summit—an international, grass-roots initiative that brings the global bioinformatics education community together for a week to work on collaborative education projects—and the second in the BioinfoCore session at ISMB—the lead annual international conference of the ISCB. At both events we invited core facility staff to join our working group. In this way, we enlisted 48 professionals from 37 institutions across 17 countries (Fig. 1A). The group has been meeting monthly since October 2023 to define roles and competency requirements. Our working documents were shared with everyone involved to enable asynchronous contributions between meetings. The work was divided into tasks, and each meeting had a distinct objective that we could achieve in the time allotted to it: we began by defining career stages; then we defined competency requirements at each stage; next we defined the Knowledge, Skills and Attitudes (KSAs) associated with the new competencies that we had defined.

To define the competencies, KSAs and career stages, the working group relied on its expertise in bioinformatics and its experience of working within core facilities, as well as referring to other related competency frameworks and recommendations for quantitative scientists and other professionals who support research. The competencies presented here align with those mentioned in the recommendations from Global Bioimaging for imaging scientist careers in core facilities (Wright *et al.* 2024). Competency frameworks developed for professionals in RIs, such as ARISE (https://competency.ebi.ac.uk/framework/arise/0.5) and RItrain (https://competency.ebi.ac.uk/framework/ritrain/1.0), have been used to define the new competencies. For example, the competency related to users’ needs reuses many elements from the ARISE framework. Frameworks and recommendations developed by communities of experts in specific areas have also provided relevant elements to the competencies presented here. For example, the skill “Advocates and negotiates for good and ethical analytical practices including integrating and resolving differing scientific approaches” derives from skills for biostatisticians (Slade *et al.* 2023). The newly developed competencies were refined and collated using the ISCB’s accepted syntax for its Competency Framework version 3.0 (Brooksbank *et al.* 2024).

A draft version of the new competencies and their associated KSAs was presented at an interactive workshop during the Global Bioinformatics Education Summit in 2024 (https://www.bioinfoedsummit.org/about-2024), in which participants were encouraged to provide feedback. There were 19 attendees from 10 countries at this session, both in-person and online. The feedback received, both in terms of wording of the competencies and in terms of how to present them to the community, was used to refine the competencies and to report on the work.

### 2.2 Survey

We designed a survey to collect input on the levels of each competency required for each of the four roles that we had defined (scientists I to III and manager).

For each role, survey respondents were asked to assign a level of competence. To simplify the task, we defined three levels of competence, aligned with Bloom’s taxonomy (Anderson and Krathwohl 2001) as follows:

- Level 1: Knowledge/Comprehension—remembering previously learned information and/or grasping the meaning of information
- Level 2: Application/Analysis—applying knowledge to actual situations and/or breaking down objects or ideas into simpler parts and seeing how the parts relate and are organized
- Level 3: Synthesis/Evaluation—rearranging component ideas into a new whole and/or making judgments based on internal evidence or external criteria

To provide some guidance, a comprehensive description was provided, including the four proposed roles, the ISCB core competency framework, and the six proposed new competencies comprising the extension to the core framework (see Supplementary Data: competency table and survey, DOI: 10.5281/zenodo.16630540). We also collected some demographic information from our survey participants, although responding to these questions was optional.

We sent the survey to members of the BioinfoCore community (bioinfo-core.org, a community of special interest of ISCB), and to participants of the 2024 Global Bioinformatics Education Summit. Taskforce members also distributed the survey to their networks via email and social media. Fifty-three individuals from 15 countries, distributed over five continents, responded.

### 2.3 Data analysis

We processed and visualized the 53 survey responses using R packages data. table v1.16.0 (Barrett *et al.* 2024), dplyr v1.1.4 (Wickham *et al.* 2023), ggplot2 v3.5.1 (Wickham 2016), tidyr v1.3.1 (Wickham *et al.* 2024), and plotly v4.10.4 (Sievert 2020) in R v4.4.1 via RStudio v2024.04.1 (R Core Team 2024). Survey responses that were “Not Applicable” were coded as zero. We modelled the career stages as a linear series to explore how these were associated with the competencies’ mean scores using Spearman’s rank correlation coefficient. We compared each competency’s correlation pattern to the mean across all competencies to identify instances where the score exceeded the 95% confidence intervals of the mean trend. We quantified the correlations of each competency pair’s scores across all four levels. The extent to which the competencies were correlated with one another was measured and visualized using principal component analysis (PCA).

**Table 1.** Manage projects.

**Knowledge** What do you need to know to exhibit competency in this area? The background and basic details of all the projects currently active within the group Project management theory and techniques The current workload of each individual and their ability to take on new projects	**Skills** What skills do you need to exhibit competency in this area? Prioritizes requests based on the biological questions involved, and selects the most important and actionable analysis to do first Prioritizes projects based on strategic importance and overlap with existing goals Estimates timelines accurately and takes reasonable steps to meet project deadlines Proactively maintains communication with end-users, providing regular updates Completes work within the allocated timespan, or proactively identifies over-run and brings it to the attention of the appropriate team member Follows expected processes and guidelines to produce high quality and reproducible work Assesses any risks that arise in the project and manages them so that the project can progress
**Effective attitudes** How does a person with this competency behave? Seeks (or gives) advice from others regarding the project direction Takes appropriate initiative on own project responsibilities Seeks guidance for project-related tasks and analyses when needed Regularly updates appropriate team members regarding progress and issues with the project Is willing to aid in projects that are at risk of going over-budget to minimize impact to clients	**Ineffective attitudes** How does a person with this competency avoid behaving? Has no sense of ownership or urgency in managing projects or missing deadlines

## 3 Results

### 3.1 Competencies added to the ISCB competency framework

The work presented here forms part of an ongoing ISCB project to develop and maintain competency requirements for bioinformatics professionals (Brooksbank *et al.* 2024), including to support career progression. In this case, the focus is placed on professional roles within bioinformatics core facilities. The 13 competencies included in the ISCB competency framework are all considered relevant for these professionals. These competencies are related to four main areas: bioscience (A–C), data science (D–F), computer science (G–I), and professional conduct (J–M).

In addition to these 13, we added six new competencies (Tables 1–6). The Supplementary Data (DOI: 10.5281/zenodo.16630540) provides the full set of competencies, including the knowledge, skills, and effective attitudes that comprise each competency. Ineffective attitudes are included, for consistency with the ISCB competency framework. The inclusion of ineffective attitudes can elicit a mixed response from users of competency frameworks: supporters of them consider them important guidance on behaviors to avoid if they wish to excel in their roles; opponents of them find their negative connotations unhelpful. We have retained them for completeness, with the caveat that those who find them unhelpful are very welcome to gloss over them.

**Table 2.** Manage team members.

**Knowledge** What do you need to know to exhibit competency in this area? Staff's individual goals to align their personal development with what the group needs People management theory and techniques, including, but not limited to, giving feedback, managing conflict, performance management and motivation of staff The competencies and values of people likely to be successful in the role Institutional policies and relevant contacts in the organization (e.g. HR, budget office, grants office)	**Skills** What skills do you need to exhibit competency in this area? Meets regularly with team members to discuss current projects, troubleshoot, hear concerns, and give and receive feedback Encourages and facilitates team building and bonding Manages or monitors all aspects of time and project management relating to staff effort, and goal timelines Writes individual development plans using SMART goals, including the identification of technical and personal development opportunities Recruits and interviews new staff Addresses issues that affect the functional group and helps management team determine plans for improvement Delegates appropriate but challenging leadership tasks to other members
**Effective attitudes** How does a person with this competency behave? Gives clear and accurate feedback, both positive and negative Tailors level and style of management to each team member Makes themselves accessible to other team members through multiple channels Resolves conflicts in a professional and unbiased manner Actively leads and participates in creating solutions to problems within the team Respects work/life balance of team members	**Ineffective attitudes** How does a person with this competency avoid behaving? Is too busy with other tasks or unavailable to assist team members Micromanages the members of the team Favors some members of the team over others Interferes in personal affairs (too intrusive)

**Table 3.** Identify and support users’ needs.

**Knowledge** What do you need to know to exhibit competency in this area? Broad overview of users' research interests and methods User experience principles applied to the core facility services Customer support practices Relationship management theory and practice Fundamentals of impact assessment The service context in which the core facility is embedded, including other core facilities and how they relate to each other.	**Skills** What skills do you need to exhibit competency in this area? Manages customer needs, differentiates wants from needs, and deals with unreasonable expectations effectively and in time Sets clear expectations on the potential and limitation of the service provided, taking into account the available resources. Systematically collects user feedback and incorporates this to improve the customer experience and increase satisfaction with the service Makes outputs (data, code, etc.) accessible to the client in a format that they can make full use of. Develops documentation to guide users or collaborators on how to interact with the core facility
**Effective attitudes** How does a person with this competency behave? Seeks to minimize assumptions regarding customer needs, problems and expectations Pursues opportunities to engage with users and understand their needs, goals, strategies, frustrations, and feedback Empathizes with users, considering the possibility that low uptake of a service might be an issue with the service and not an issue with the users	**Ineffective attitudes** How does a person with this competency avoid behaving? Prioritizes core facility needs over clients (e.g. designs processes to make their own lives easier at the expense of usability) Favors some customers (friends, ex-colleagues, etc.) beyond scientific and strategic objectives

**Table 4.** Engage with users and other collaborators.

**Knowledge** What do you need to know to exhibit competency in this area? Current techniques, analysis methods and theories in the collaborators’ domain of expertise The different scales of projects and the implications of this to resourcing projects Team science and its application, such as identifying gaps in expertise and how to fill them	**Skills** What skills do you need to exhibit competency in this area? Engages regularly with collaborators, keeping them updated on progress and seeking input on next steps Manages expectations by clearly defining the work to be done within time and budget Monitors opinions about the core through informal communication and/or surveys; Empathizes with collaborators to understand their motivation and potential challenges Works effectively with a broad range of collaborators Advocates and negotiates for good and ethical analytical practices including integrating and resolving differing scientific approaches Resolves conflicts, taking into account the needs of all the parties involved
**Effective attitudes** How does a person with this competency behave? Welcomes people to interact with the core facility, for example through training, journal clubs, seminars and surgeries Helps people with small questions or problems Welcomes researchers’ ideas and contributions to their project Is open to collaborate with a broad range of professionals Clarifies the expectations of collaboration versus service (e.g. regarding authorship)	**Ineffective attitudes** How does a person with this competency avoid behaving? Performs analyses that are not relevant, useful or interesting to the collaborator Refuses to accept ideas from the collaborators in relation to the analysis Agrees to unrealistic requests instead of having honest conversations about the core facility’s capacity Does not see the value in proactively managing relationship with clients

**Table 5.** Provide training in bioinformatics.

**Knowledge** What do you need to know to exhibit competency in this area? How different pedagogical approaches meet learners needs The topic being taught Principles of course and program design Effective approaches to active learning in hybrid and virtual learning environments How to apply constructive alignment that lacks direct assessment methods FAIR principles, the data lifecycle and their application to training	**Skills** What skills do you need to exhibit competency in this area? Provides attendees with course materials aligning with FAIR principles and the data lifecycle Chooses learning activities supporting learning outcomes Uses appropriate technologies and datasets for each class Adapts training based on learners’ progress Assesses and measures learning outcomes and program outcomes Acts on feedback to improve future courses Acknowledges formally users’ long-term training achievements
**Effective attitudes** How does a person with this competency behave? Flexibility to meet the diverse needs of learners in mixed classes Champions and advocates for the benefits of training for learners Monitors demand for new courses and emerging topics to enhance current courses Nurtures a bioinformatics community of practice Considers diversity, equality, inclusion and accessibility (DEIA) to create content and events that meet the needs of all learners Ensures that learners can apply their new competencies to tasks relevant to their roles Priorities class time spent on fundamentals and misconceptions ahead of transient software tools	**Ineffective attitudes** How does a person with this competency avoid behaving? Shows expert bias, i.e. assumes that learners have skills that are obvious for an expert Uses jargon that not everyone can understand Considers training a burden Views research as more important than education Focuses on advanced knowledge/skills rather than targeting foundational topics

**Table 6.** Lead a bioinformatics core facility.

**Knowledge** What do you need to know to exhibit competency in this area? Fundamentals of leadership and the difference between leadership and management The current status of the bioinformatics field The areas of research at their institution Cultural awareness and its application in a leadership context Fundamentals of business development Fundamentals of quality control and quality assurance	**Skills** What skills do you need to exhibit competency in this area? Sets long-term strategic goals and uses them to prioritize the facilities activities and investments Identifies areas for the group to improve and directs implementation of changes Develops and manages new collaborations with research groups and establishes good working relationships with other core facilities Manages the relationship with clients Ensures that income stream is sufficient to sustain the facility Ensures that the facility has appropriate visibility for its size and its client base Forges appropriate strategic alliances, collaborations and business agreements
**Effective attitudes** How does a person with this competency behave? Is open to 360° feedback, both positive and negative Supports managers and supervisors in overseeing early-career staff Participates actively in attracting new projects and users, for example through networking, managing relationships, initiating projects, and being the ‘public face’ of the facility Performs horizon scanning to identify new developments that will impact the work of the facility Promotes the positive impact that the facility has on its institute and in the wider context	**Ineffective attitudes** How does a person with this competency avoid behaving? Manages the team in an overly authoritarian way Focuses exclusively on technical work instead of on leadership Lacks a long-term vision for the facility

The new competencies cover (but are almost certainly not specific to) the abilities and responsibilities required in a bioinformatics core facility. They are: identify and support users’ needs (labelled N1; defined fully in Table 1), manage projects (O1; Table 2), manage team members (P1; Table 3), engage with users and other collaborators (Q1; Table 4), provide training in bioinformatics (R1; Table 5), and lead the bioinformatics core facility (S1; Table 6). These competencies reflect the nature of the work performed in bioinformatics core facilities, where there are a variety of projects with collaborating researchers. Two of the six new competencies are related to management, one focusing on project management, the other on people management. Three are related to the relationship between the staff at the facility and others: one focuses on supporting user needs, another on engagement with collaborators of the facility and the third on training. The latter is typically targeted beyond immediate users or collaborators to reach researchers in their institution and/or local network. The final competency includes the knowledge, skills, and attitudes essential for leading a core facility. This leadership competency spans a large spectrum of responsibility, which varies with the size and context of a facility. For example, a large, well-established facility might have a leader whose entire focus is on leading the team. By contrast, a small (or new) facility might have a leader who needs to balance leadership with technical tasks. As with all the roles associated with the ISCB competency framework, we define the minimum level of competency required.

While additional competencies are clearly required as core facility scientists progress through their careers, others might become less important and might no longer be emphasized in selecting individuals for progression into managerial and leadership roles. We did not capture this in the extended framework, because the context of an individual role in a specific core facility is all-important. Technical leadership versus people leadership, for example, requires weighting towards different competencies: someone progressing towards leadership of a large team might focus on developing competency L3: “Work effectively in teams to accomplish a common goal” to a high level, and only need to retain a broad overview of competency I3: “Construct, manage, and maintain bioinformatics computing infrastructure of varying complexity.” Someone progressing to technical leadership, perhaps in a setting where access to compute is not provided by the host institute, might need to retain and refresh their IT infrastructure competency as a high priority. Furthermore, managers of small core facilities often remain responsible for delivering on a proportion of the technical workload; these managers find it necessary to retain and update both their technical and their professional-conduct competencies. For these reasons, we focused more on the need to gain new competencies or enhance existing ones, and less on the likelihood of technical competencies becoming less important as facility members become more senior. If, in the future, it becomes helpful to differentiate the roles further, we might consider weighting some competencies accordingly.

Each competency includes KSAs required for a person to be considered competent in that area (Tables 1–6). The number of elements in each knowledge (K), skill (S), or attitude (A) attribute can vary from 1 to 7, in line with the range agreed upon by the ISCB Careers and Competency Taskforce during the development of version 3.0 of the ISCB Competency Framework (Brooksbank *et al.* 2024). The KSAs are written in a way that is concrete enough for professionals to relate to the statements and associate activities relevant to their roles with them, yet broad enough to be applicable in a variety of contexts. The ISCB Competency Framework is viewed as a minimum standard, and an individual must possess all the KSAs listed (except the ineffective attitudes) to be considered to have met the competency requirement. These same principles hold for the new competency statements, and drove our desire to cap the number of attributes at a manageable figure.

### 3.2 Reflecting career progression for bioinformatics core facility scientists

Career progression has clearly defined steps for researchers in academia, from a PhD through one or more postdoctoral fellowships to a principal investigator position. By contrast, there is no well-defined career path for professionals working in bioinformatics core facilities. We established four career profiles to provide a structure that can guide facilities and professionals. These include three roles for core facility scientists: entry level (I), mid-level (II), high level (III) with increasing levels of experience and responsibility, and a managerial role (IV) capturing the competencies required by core-facility leaders (including leaders of teams embedded within large core facilities).

**Scientist I** is an entry-level professional who is building foundational expertise across multiple technical and computational competencies. They contribute to bioinformatics workflows, prepare and manage data, and support research activities, while developing their skills in communication, teamwork, and ethical conduct. Scientist I roles typically focus on applying established methods and collaborating with others to accomplish project goals.

**Scientist II** is an experienced practitioner with solid technical knowledge and the ability to independently perform complex tasks in bioinformatics. They are proficient in data analysis, scripting, and tool use, and often contribute to the development and improvement of resources. Scientist II staff might begin to manage small projects or supervise team members, engage more deeply with collaborators, and provide some training or user support.

**Scientist III** is an expert in bioinformatics, demonstrating mastery in technical domains, data science, and multi-faceted research challenges. They regularly drive scientific discovery, lead the development of user-centric tools, and manage both projects and people. Scientist III professionals are effective communicators and mentors, who contribute significantly to advancing the goals of a core facility and participate in the broader scientific community.

**Manager** oversees the strategic direction and operational leadership of their bioinformatics core facility. Managers ensure excellence across all competencies, from technical depth to team management, training, and project leadership. They are responsible for guiding their team(s), stewarding resources, fostering innovation, and engaging with diverse stakeholders to deliver impactful bioinformatics solutions for their organization and perhaps also for a broader community.

Core facilities have varying sizes and levels of complexity, so our intention is that this scheme is used in a flexible manner for each facility, according to its needs. For example, some facilities might function well with two scientist levels and a manager; others might require a deeper hierarchy. Some institutions have their own competency frameworks for staff. In such cases, aligning the two might help managers and human resources teams to come to consensus on which competencies are most relevant for each role in that context. The framework presented here is intended to provide a foundation on which any bioinformatics core might build according to its needs.

The competency framework allows us to define the knowledge, skills, and attitudes required at each career stage. To do this in a way that reflects the views of the bioinformatics core facility community, we created and distributed a survey in which respondents could evaluate the levels of competency required for each competency statement and for each of the four professional stages that we had defined. We obtained 53 responses from 15 countries distributed over five continents (Fig. 1B) Respondents assigned increasing levels of competency as bioinformatics professionals progress from scientist I to scientist III. This continues to increase for a subset of the competencies at the managerial stage (Table 7).

**Table 7.** Summary of levels of competency required at the four defined career stages^a^.

Competency area	ID	Competency	Scientist I	Scientist II	Scientist III	Manager
Bioscience	A3	Work at depth in at least one technical area aligned with the life sciences	1	2	3	3
Bioscience	B3	Prepare life science data for computational analysis	2*	2	3	3
Bioscience	C3	Have a positive impact on scientific discovery through bioinformatics	1	2	3	3
Data science	D3	Use data science methods suitable for the size and complexity of the data	1	2*	3*	3
Data science	E3	Manage own and others’ data according to community standards and principles	2*	2	3*	3
Data science	F3	Make appropriate use of bioinformatics tools and resources	2*	3*	3*	3
Computer science	G3	Contribute effectively to the design and development of user-centric bioinformatics tools and resources	1	2	3	3
Computer science	H3	Make appropriate and efficient use of scripting and programming languages	2*	3*	3	3
Computer science	I3	Construct, manage and maintain bioinformatics computing infrastructure of varying complexity	1	2	3	3
Professional conduct	J3	Comply with professional, ethical, legal and social standards and codes of conduct relevant to computational biology	1	3*	3	3
Professional conduct	K3	Communicate meaningfully with a range of audiences - within and beyond your profession	1	2	3	3
Professional conduct	L3	Work effectively in teams to accomplish a common goal	2*	3*	3*	3
Professional conduct	M3	Engage in continuing professional development in bioinformatics	1	3	3	3
Core facility-centric	N	Manage projects	2*	2	3	3
Core facility-centric	O	Manage team members	0	2	3	3*
Core facility-centric	P	Identify and support users' needs	0	0	2	3
Core facility-centric	Q	Engage with collaborators	1	2	3	3
Core facility-centric	R	Provide training in bioinformatics	1	2	3	3
Core facility-centric	S	Lead the bioinformatics core facility	0	0	2	3

a The numbers in each field refer to the competency level required, where level 0 signifies no competency required; level 1 signifies knowledge/comprehension (remembering previously learned information and/or grasping the meaning of information); level 2 signifies application/analysis (applying knowledge to actual situations and/or breaking down objects or ideas into simpler parts and seeing how the parts relate and are organized); and level 3 signifies synthesis/evaluation (rearranging component ideas into a new whole and/or making judgments based on internal evidence or external criteria). The fields marked with an asterisk are those in which analysis of the survey results revealed a confidence interval (CI) of at least 95%. The Competencies from version 3 of the ISCB Competency Framework are labeled A3 through M3; the new competencies designed for bioinformatics core facility managers are labeled N through S.

**Figure 2.**
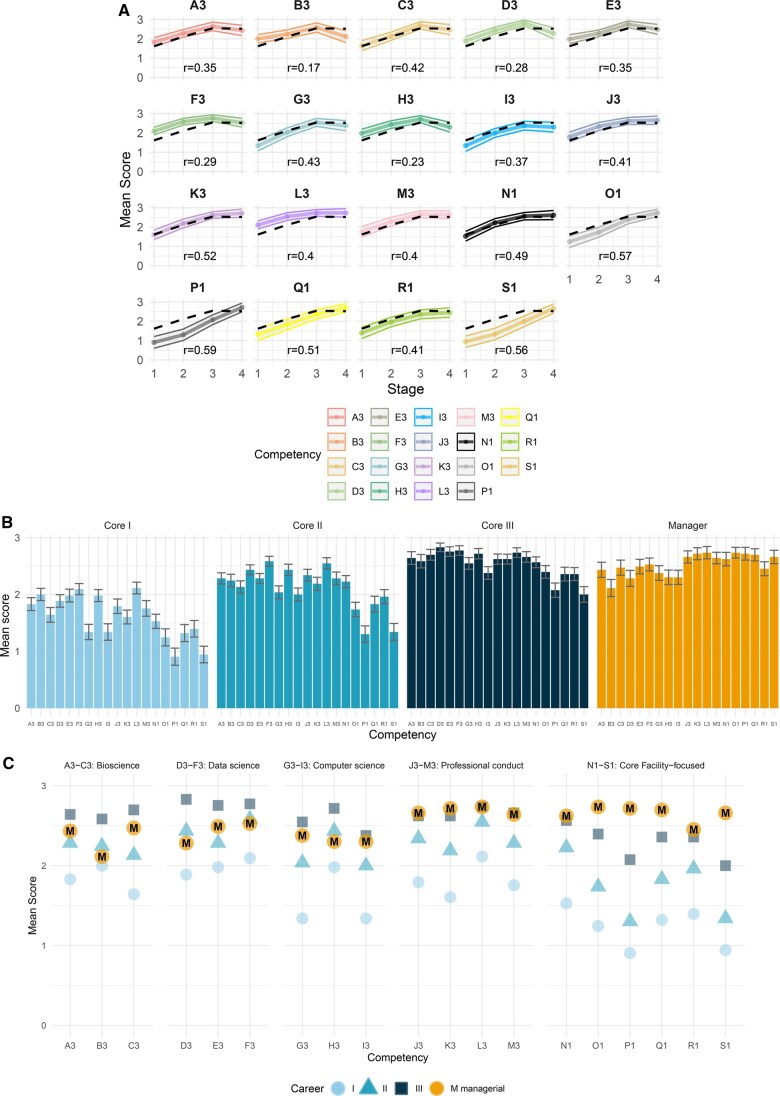
The competencies’ mean survey scores. (A) The correlation of career stage (*x*-axis) with mean competency score. The *r* values denote Spearman’s rank correlation coefficient for each competency. The thin colored lines on each plot show the 95% confidence intervals. The black dashed line is the average correlation pattern across competencies. (B) Bar plots display the mean score for each competence across the four career stages. Error bars indicate the standard error. (C) Competencies’ mean scores for each career stage. Scientist I is shown by circles, scientist II by triangles, scientist III by squares, and manager by circles labelled ‘M’ . The competencies are grouped into those related to bioscience (A3–C3), data science (D3–F3), computer science (G3–I3), professional conduct (J3–M3), and the core-facility-focused competencies proposed by this study (N1–S1).

We compared each competency's correlation pattern to the mean across all competencies to identify instances where the score exceeded the 95% confidence intervals of the mean trend (Fig. 2).

For scientists at stage I, of the nine technical competencies (A3 through I3) in version 3.0 of the ISCB Competency Framework, four of them (B, E, F, and H) were consistently reported (with a CI of >95%) as being required at level 2 (Application/Analysis). These were:

- B3: Prepare life science data for computational analysis
- E3: Manage own and others’ data according to community standards and principles
- F3: Make appropriate use of bioinformatics tools and resources, and
- H3: Make appropriate and efficient use of scripting and programming languages.

Survey respondents also noted that two of the competencies related to professional conduct, work effectively in teams (L3) and manage projects (N), are also necessary.

For scientists at stage II, the survey results revealed a CI of >95% for development of the following competencies:

- D3: Use data science methods suitable for the size and complexity of the data
- F3: Use data science methods suitable for the size and complexity of the data (increase from level 2 to level 3, Synthesis/Evaluation),
- H3: Make appropriate and efficient use of scripting and programming languages (increase from level 2 to level 3, Synthesis/Evaluation)
- J3: Comply with professional, ethical, legal and social standards and codes of conduct relevant to computational biology
- L3: Work effectively in teams to accomplish a common goal (increase from level 2 to level 3)

For scientists at stage III, the survey results revealed a high CI score for development of the following competencies to level 3:

- D3: Use data science methods suitable for the size and complexity of the data
- E3: Manage own and others’ data according to community standards and principles
- F3: Make appropriate use of bioinformatics tools and resources
- L3: Work effectively in teams to accomplish a common goal

Finally, for the managerial stage, the survey results revealed a Cl score of >95% for project management (O) at competency level 3.

For 17 out of the 19 competencies, there were no large differences between managerial and scientist III competency requirements; most are at level 3 for both roles. Survey respondents considered a higher level of competency in managers to be a requirement for only two competencies: “Identify and support users’ needs” (P1) and “Lead the bioinformatics core facility” (S1).

Our survey responses supported the addition of the six new competencies added in this framework (N1–S1), whose scores were higher for the scientist III and managerial stages, and notably lower for the scientist I and II stages (Table 7, Fig. 2A). This pattern was weaker for the new competency related to training (R), which reflects that training may be delivered by early and mid-career core facility staff. Among the existing 13 competencies, only communication (K) showed a clear expectation of a higher level in line with the career stage.

Our work started with the ISCB competency framework, which focuses on generic competencies in computational biology, and not specifically in bioinformatics core facilities. We sought to add new relevant competencies absent in the generic ISCB framework, resulting in six new competencies that were widely acknowledged as a requirement for core facility scientists at all career stages, and even more notably for core facility managers, based on our survey responses.

We examined the importance of each competency across career stages and summarized this using the mean survey scores per competency and stage (Fig. 2A–C). In line with the above, we observed an increased competency requirement in the more senior roles for the competencies added in this work (N1-S1), together with three more related to transversal competencies: communication (K3), working in teams (L3) and engaging in professional development (M3).

**Figure 3.**
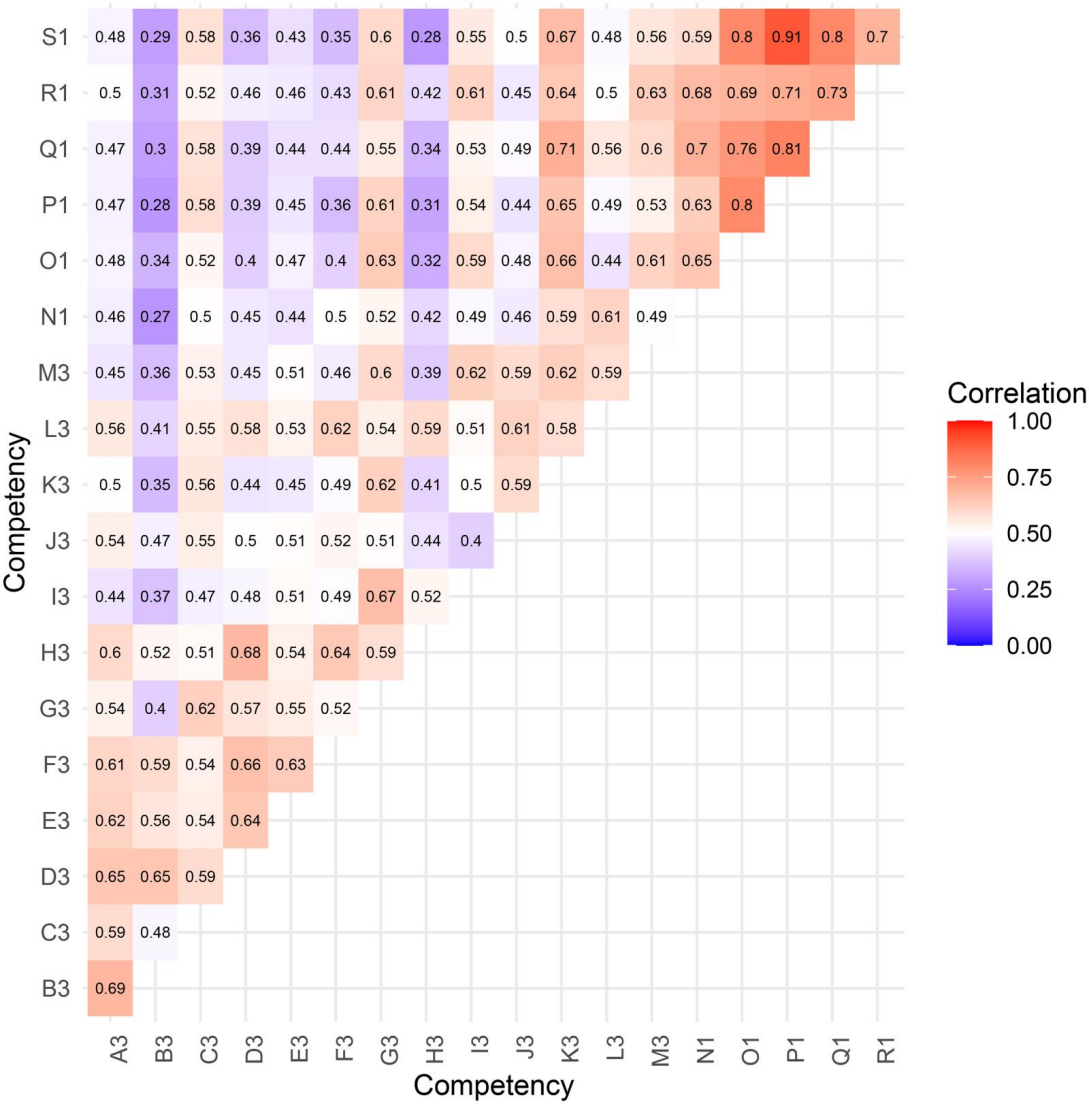
The pairwise correlations between competencies based on Spearman’s rank correlation coefficient. The range is from 0 (blue) to 0.5 (white) to 1.0 (red). The correlation values are shown for each pair.

A correlation analysis (Fig. 3) was performed to determine whether the survey revealed any strong positive or negative correlations between pairs of competency requirements. The two competencies whose scores were less correlated with the other competencies are technical ones: B3 (Prepare life science data for computational analysis) and H3 (Make appropriate and efficient use of scripting and programming languages). The correlations between our six new competencies, designed specifically with core facility scientists in mind, tended to be higher.

**Figure 4.**
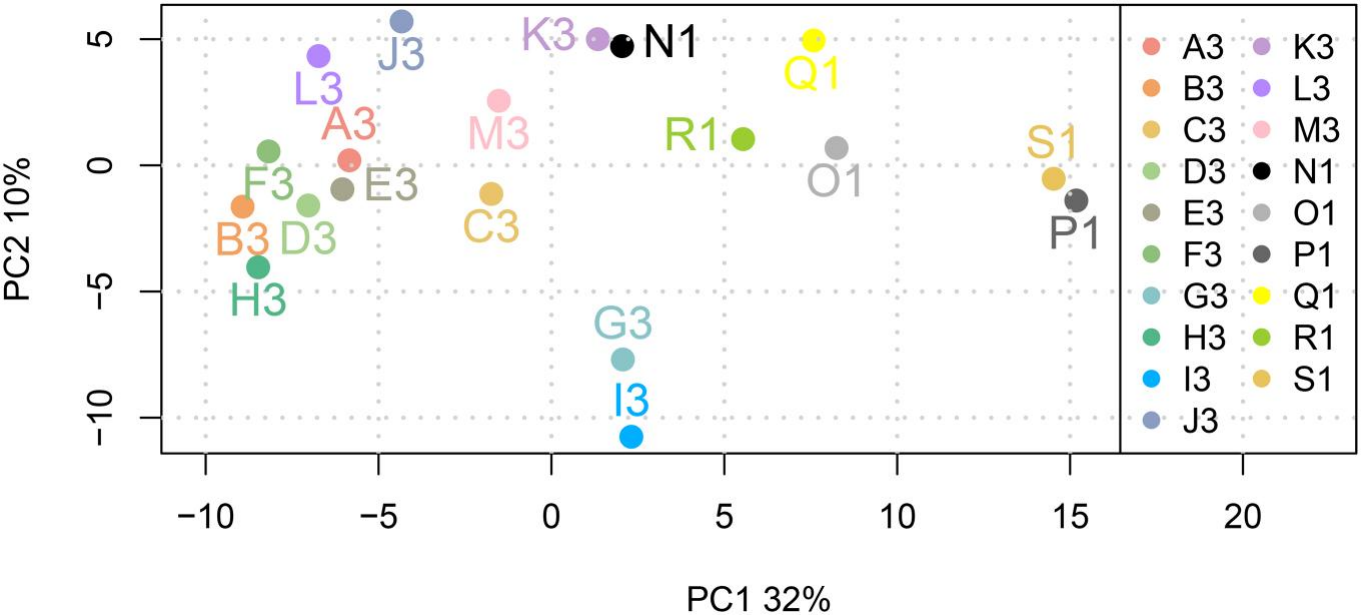
Principal component (PC) analysis of the correlations across the competency data shown across PC1 (accounting for 32% of all variation) and PC (accounting for 10% of all variation).

We explored the extent to which the competencies were correlated with one another using PCA (Fig. 4). This revealed that two of the computer science competencies, G3 (Contribute effectively to the design and development of user-centric bioinformatics tools and resources) and I3 (construct, manage and maintain bioinformatics computing infrastructure of varying complexity) correlated relatively weakly with the other competencies. Whilst some core facilities do contribute to, and might even lead, the development of user-centric tools and resources, this has increasingly become the domain of large-scale RIs, serving researchers on a national or international level (for example, the Swiss Institute of Bioinformatics, SciLifeLab and the Indian Biological Data Centre; the European Bioinformatics Institute, NCBI, and DDBJ).

With the rapid development of computing infrastructure, which now can include cloud computing in addition to local institutional clusters, and with the elevated focus on cybersecurity, responsibility for cyberinfrastructure is shifting towards the IT department in the institution that hosts the core facility. Nevertheless, some core facilities do maintain their own cyberinfrastructure.

Bioinformatics core facilities provide bioinformatics services to others. The nature of this work is clearly reflected in the newly added competencies, with a focus on workload management, people management and relationship building with users or collaborators. It’s interesting to note that many survey respondents consider that managers require a high level for all competencies, which might not be maintainable as senior members of a facility gain leadership responsibilities and become less directly involved in technical aspects of their team members’ work; this might reflect the variable nature of bioinformatics core facilities, in terms of both size and governance within their institution. In smaller core facilities, managers often find it necessary to retain a proportion of the technical workload and therefore do need to retain—and continue to develop—their technical competency. Indeed, the ISCB core competency M3: “Engage in continuing professional development in bioinformatics,” was considered necessary at level 3 in all career stages except stage 1, entry level.

### 3.3 How organizations can use the framework

Successful core facilities in bioinformatics recognize that effectively hiring, training, managing, and retaining the best bioinformaticians possible is one of the most important things they can do to maintain and build upon their success. Technologies, programming languages, and approaches will change, but projects progress, and science advances, through the contribution of bioinformaticians who are innovative, collaborative, and good at solving problems. While this framework is not intended to be a universal set of competencies that will apply directly to every core facility, it does provide a starting point to help cores develop customized job descriptions, and enables them to offer a clearer and more systematic career progression pathway to their bioinformaticians. Different cores have different structures and might need to emphasize distinct competency requirements. Cores can use this framework when writing job postings to recruit at a particular level. They can also use it during performance reviews to determine when individuals are ready to progress to the next level, and to support them to work towards more senior roles. Managers of new cores can use it to determine the knowledge, skills, and attitudes they need to look for in job applicants.

### 3.4 How individuals can use the framework

Bioinformatics professionals in core facilities are often drawn to the field from diverse backgrounds. Since this is a relatively new and interdisciplinary career, it may not always be clear what knowledge, skills, and attitudes are needed to advance professionally. Individuals can evaluate their own competency in each area against the framework and decide where they might benefit from investing their effort. The framework can be used to steer individuals towards developing new competencies that might help them to advance down their chosen career path. Such self-evaluation might also be an entry point to a conversation with their managers regarding promotion or what steps they need to follow to get to the next level of their career.

## 4 Conclusions and perspective

In this work, we extended the ISCB competency framework to define competencies that are especially relevant for professionals in bioinformatics core facilities, with the aim of providing structure and guidance to both institutions and professionals on the definition of career pathways. The work was a community effort involving two ISCB communities of scientific interest (COSIs)—BioInfoCore and Education—and open to other professionals interested in the initiative. We hope that other communities within or beyond the ISCB might find it helpful to use or adapt the approach that we have taken here to define minimum competency requirements at different career stages for their own professions.

Competency frameworks are not static; they need to evolve to remain useful for professionals in a world that is in constant and rapid change, especially with the fast speed of advancements in high-throughput analysis, computational power, and artificial intelligence. The use of the ISCB competency framework across bioinformatics core facilities will inform its future developments to adapt to changes in the profession. Areas to watch include advanced statistical skills such as those used in artificial intelligence, and the considerable demands on core facilities posed by spatial multiomics. With regards to the creation of “hybrid” career paths, incorporating bioinformatics into diverse fields, we hope that we have defined a viable, pragmatic, and community-based method for others to use and adapt as needed. The core ISCB competency framework will need to continue to evolve with the field, and it is possible that some of the competencies already defined might shift, from one version to the next, from core to a specific profession and back. It is up to the ISCB community to agree on how relevant each competency requirement is to each context that it considers important to define a professional development path for.

As the field of bioinformatics continues to expand and evolve, bioinformatics cores often operate in relative isolation. However, there are significant commonalities in our experiences that provide opportunities for mutual learning and collaboration. Our work illustrates that transversal and managerial skills are as important as technical attributes to the successful operation of bioinformatics core facilities. Through the development of this extension to the core ISCB competency framework, we aim to systematically define the competencies required by core facility professionals, enhance the recruitment, and retention of highly qualified individuals in a dynamic and intellectually stimulating field, and strengthen the bioinformatics capabilities necessary to advance biomedical research in the 21st century, thus contributing towards addressing global sustainable development challenges as alluded to in the Brisbane Statement.

## Data Availability

The framework is reproduced in full in this paper and is available as a CSV file on Zenodo (DOI: 10.5281/zenodo.16630540). It will soon be available on the Competency Hub at https://competency.ebi.ac.uk/. Data and figures are available on GitHub at https://github.com/downingtim/Competencies.
